# A comparative study of blood endotoxin detection in haemodialysis patients

**DOI:** 10.1186/s12950-016-0132-5

**Published:** 2016-07-30

**Authors:** Jonathan Wong, Nathan Davies, Hasan Jeraj, Enric Vilar, Adie Viljoen, Ken Farrington

**Affiliations:** 1 Department of Renal Medicine, Lister Hospital, Corey Mills Lane, Stevenage, Hertfordshire SG1 4AB UK; 2 University of Hertfordshire, College Lane, Hatfield, AL10 9AB UK; 3 UCL Institute for Liver and Digestive Health, Royal Free Hospital, Pond Street, London, NW3 2QG UK; 4 Quality Control Department, Lister Hospital, Corey Mills Lane, Stevenage, Hertfordshire SG1 4AB UK; 5 Department of Biochemistry, Lister Hospital, Corey Mills Lane, Stevenage, Hertfordshire SG1 4AB UK

**Keywords:** Endotoxin, Haemodialysis, Inflammation

## Abstract

**Background:**

Endotoxemia is commonly reported in patients receiving haemodialysis and implicated in the pathogenesis of systemic inflammation. The Limulus Amoebocyte Lysate (LAL) assay is the most commonly used blood endotoxin detection assay. Two kinetic variations of the assay are commercially available – the turbidimetric and chromogenic assay, it is unknown which assay is superior for endotoxin detection in uremic patients. Selection of the optimum LAL technique for endotoxin detection in haemodialysis patients is important to further understanding of the sequela of endotoxemia and development of endotoxin-lowering strategies in this population.

**Method:**

A turbidimetric and chromogenic LAL assay from the same manufacturer were directly compared. We investigated the ability of both LAL assays to detect endotoxin in uremic plasma. Plasma samples from haemodialysis patients and healthy controls were spiked with endotoxin and percentage spike recovery for the chromogenic and turbidimetric assay was determined. Assay accuracy and precision were compared between both LAL assays.

**Results:**

The turbidimetric assay had greater accuracy than the chromogenic assay. Spike recovery was 113.8 % vs. 53.8 % for the turbidimetric and chromogenic assay respectively. Assay bias was higher in the chromogenic assay (−0.384EU/mL vs. 0.011EU/mL). The turbidimetric assay demonstrated greater precision compared to the chromogenic assay. Coefficient of variation ranged from 4.5 to 24.1 % for the turbidimetric assay and 25.8–26.5 % for the chromogenic assay.

**Conclusion:**

The study findings suggest that the kinetic turbidimetric LAL assay has greater accuracy and precision than the chromogenic assay and is the optimum LAL technique for endotoxin detection in haemodialysis patients, though these findings should be verified using LAL reagents from other sources.

## Background

Endotoxemia is widely reported phenomenon in haemodialysis (HD) patients [1, 2], however endotoxin detection in blood is difficult and the optimal assay for use in HD patients is unknown. Many different endotoxin detection assays have been employed in previous studies in dialysis patients, including the turbidimetric Limulus Amoebocyte Lysate (LAL) assay, chromogenic LAL assay, Endotoxin Scattering Photometry (ESP) and Endotoxin Activity Assay (EAA) [2]. It is important to determine the optimum detection assay for use in patients with end-stage kidney disease (ESKD) since endotoxemia is reported to be associated with chronic inflammation [3] – itself a poor prognostic marker [4]. Accurate endotoxin measurements are essential to further understanding of the sequela of endotoxemia in this population and to facilitate the development of potential endotoxin-lowering strategies.

The LAL assay is the most commonly used endotoxin detection assay. LAL is derived from extracts of primitive amoebocytes present in the haemolymph of the horseshoe crab. The lipid A component of endotoxin interacts with pro-clotting enzymes present in LAL activating a coagulation cascade resulting in gelation and eventual clot formation [5]. High endotoxin content samples lead to rapid gelation. The rate of increase in turbidity can be measured using a spectrophotometer to quantify the endotoxin content of a sample. This technique is known as the turbidimetric LAL assay. A variation of this technique involves the addition of chromogenic substrates to LAL which undergoes a colour change in the presence of endotoxin. The chromogenic LAL assay has been the most commonly used endotoxin detection method in previous human studies and dialysis literature [2, 6]. However, in Japan, the turbidimetric LAL assay is routinely used and is covered by the Japanese national health insurance program [7].

We recently demonstrated that a specific commercially available kinetic turbidimetric Limulus Amoebocyte Lysate (LAL) was an accurate and precise endotoxin detection assay in HD patients [8], although it is not known whether LAL assays utilising chromogenic techniques may have better performance since direct comparative studies in ESKD patients have not been published. Using the same study subjects [8], we carried out endotoxin spike recovery experiments to directly compare the accuracy and precision of a turbidimetric LAL with a chromogenic LAL assay from the same manufacturer.

## Methods

### Participant selection

The whole study cohort consisted of seven clinically stable patients undergoing outpatient HD (mean age 63) at the Lister Renal Unit (Hertfordshire, UK) and seven healthy controls (mean age 47) recruited from volunteers and healthcare workers at the Lister Hospital (Hertfordshire, UK). To minimise the possibility of high amounts of native endotoxin present in plasma samples which would interfere with endotoxin spike recovery experiments, only subjects who were clinically well with no evidence of sepsis were recruited to the study. Additionally, HD patients were required to have a C-reactive protein (CRP) measurement of <5 mg/L within the last month to be eligible for recruitment. Patients with a venous catheter in-situ, liver dysfunction, congestive cardiac failure and active gastrointestinal or inflammatory diseases were not eligible for recruitment.

### Blood collection

All phlebotomy equipment including blood collection and storage tubes were checked for endotoxin contamination and interfering factors by random batch testing as previously described [8, 9]. All apparatus had no detectable endotoxin (<0.005EU/ml). Blood samples were collected aseptically into sterile Terumo Venoject II heparinized tubes (Project KBF, Tokyo) and kept chilled on ice. In HD patients, blood was sampled pre-dialysis from the arteriovenous fistula. Blood samples were centrifuged at 250 g for 10 min at 4 °C to obtain plasma and stored in Eppendorf Biopur® safe-lock tubes and immediately frozen and stored at −80 °C.

### Plasma pre-treatment

Endotoxin spike recovery in uraemic plasma samples spiked with high concentration endotoxin can be optimised by diluting samples in Tween 80 compared to the standard method of diluting in endotoxin-free water [8, 10]. Plasma samples were diluted 1:10 in 0.1 % Tween 80 (Merck Chemicals, Darmstadt, Germany) and heated at 70 °C for 10 min to denature plasma proteins and proteases which may interfere with endotoxin detection by the LAL assay [11, 12]. Batches of 0.1 % Tween 80 were consistently found to have no detectable endotoxin and were free of interfering factors (endotoxin spike recoveries ranged from 94 to 106 %). Samples were cooled to room temperature (20–25 °C) prior to endotoxin measurements.

### Assessment of assay accuracy

Plasma samples from five HD patients and five healthy controls were divided into aliquots and directly spiked with five different concentrations of control standard *E.coli 055:B55* endotoxin (0 [unspiked sample], 0.05, 0.1, 0.5 and 2.5 EU/ml) and then diluted in 0.1 % Tween 80 and subjected to heat treatment as described above. Each sample was tested for endotoxin content in sextuplicate using both the kinetic chromogenic and kinetic turbidimetric LAL assay. Samples that contained bubbles introducing artefact into optical density graphs were discarded from the analysis. All samples had between four to six repeated measurements. Percentage spike recovery was calculated for each spiked plasma sample using the formula:-

$$ \%\  spike\  recovery=\frac{Measured\  endotoxin\  content\  in\  spike d\  sample - Measured\  endotoxin\  content\  in\  unspiked\  sample}{Amount\  of\  added\  endotoxin} \times 100\% $$

The optimal spike recovery is 100 %, although a spike recovery between 50 and 200 % is considered valid according to industry guidance [13]. Assay accuracy was assessed by comparing difference in spike recovery between the chromogenic and turbidimetric LAL assay using Wilcoxon signed rank test. Differences in baseline endotoxin content in unspiked samples between the chromogenic and turbidimetric assay was compared using the Friedman test. Measured endotoxin content was compared with expected endotoxin content in spiked samples using Bland-Altman analysis [14] and linear regression analysis was used to calculate slope and y-intercept of the line of best fit to estimate magnitude of proportional and constant error for both assays [15].

### Assessment of assay precision

Samples from two HD patients and two healthy controls were pooled separately. Pooled HD and healthy control plasma were spiked with two different concentrations of control standard endotoxin (0.05 and 0.5 EU/ml), diluted and heat treated as described earlier and subjected to ten repeated assay measurements for endotoxin using the kinetic chromogenic and kinetic turbidimetric LAL assay to calculate a coefficient of variation (CV). The CV is used to assess assay precision [16] but there is no industry guidance on a maximum acceptable CV, although most LAL manufacturers impose a CV <10–20 % for results to be considered valid [16].

### Endotoxin assays

*Kinetic turbidimetric LAL assay:* samples were assayed using Endosafe KTA2 lysate (Charles River Laboratories, France) on sterile 96-well microplates (manufacturer certified to <0.001EU/ml). Assays were carried out using manufacturer supplied equipment including depyrogenated glass tube, pipettes and Eppendorf Endosafe pipette tips (certified <0.005 EU/ml). Analysis of each microplate consisted of duplicate wells containing endotoxin-free water to act as a negative control. Plates were analysed using a Biotek ELx808 absorbance microplate reader with Endoscan-V software (version 4.0; Charles River Laboratories, France) and observed at 340 nm with an onset optical density value set at 0.03 as per manufacturer’s recommendations. Six-point standard curves were constructed using onset reaction times from standard dilutions of control standard endotoxin (*E.coli* 055: B5) ranging from 10 to 0.0025 EU/ml. All standard curves constructed had a correlation coefficient of >0.98, as required for valid extrapolation of reaction times [13]. Due to the ten-fold dilution used for plasma, the lower limit of detection was 0.025 EU/ml.

*Kinetic chromogenic LAL assay*: samples were assayed using Endochrome-K lysate (Charles River Laboratories, France) with manufacturer supplied depyrogenated glass tubes, pipettes and pipette tips as described above. Microplates were analysed using FLUOstar Omega microplate reader with MARS data analysis software (BMG Labtech, Offenburg, Germany) and observed at 405 nm with an optical density value of 0.1 as per manufacturer’s recommendations. The same concentrations of control standard endotoxin were used to construct standard curves as described for the kinetic turbidimetric LAL assay.

## Results

### Comparison of assay accuracy between turbidimetric and chromogenic LAL assay

This sub-study consisted of five HD patients and five healthy controls. In unspiked plasma samples, the kinetic turbidimetric LAL assay detected endotoxemia in four out of five HD patients whereas the kinetic chromogenic LAL assay detected endotoxemia in one out of five HD patients (median endotoxin level 0.041 EU/ml [IQR 0.016–0.081]] vs. 0 EU/ml [IQR 0–0.017]; *p* < 0.001). In healthy controls, only one subject had detectable endotoxemia (0.027 EU/ml) using the turbidimetric assay. No healthy subjects had detectable endotoxemia using the chromogenic assay.

Spike recovery was within for the industry specified 50–200 % [13] range for all levels of spike using the turbidimetric assay for both HD patients and healthy controls. Spike recovery with the chromogenic assay was significantly lower than the turbidimetric assay for HD patients (53.8 % vs. 113.8 %; *p* < 0.001) and healthy controls (34.0 % vs. 114.0 %; *p* < 0.001) (Table 1). Notably, for the chromogenic assay spike recovery was frequently below the minimum required spike recovery of 50 % suggesting persistent inhibition of the chromogenic LAL assay by interfering factors present in HD and healthy plasma despite dilution and heat-treatment.

**Table 1:** Comparison of spike recovery of turbidimetric and chromogenic LAL assay

Concentration of endotoxin spike (EU/ml)		0 (unspiked sample)	0.05	0.1	0.5	2.5	Overall
Population							
HD patients (*n* = 5)	Chromogenic assay						
	Endotoxin content (EU/ml) of sample	0 [IQR 0–0.017]	0.026 [IQR 0–0.049]	0.074 [IQR 0.033–0.1]	0.242 [IQR 0.198–0.331]	1.69 [IQR 1.492–1.859]	-
	Spike recovery (%)	-	15.3 [IQR −7.3–97.6]	40.2 [IQR 32.8–100.4]	46.4 [IQR 39.6–63.8]	67.6 [IQR 59.7–73.7]	53.8 [IQR 34.0–78.9]
	Turbidimetric assay						
	Endotoxin content (EU/ml)	0.041 [IQR 0.016–0.081]	0.109 [IQR 0.092–0.118]	0.176 [IQR 0.126–0.205]	0.525 [IQR 0.408–0.817]	2.715 [IQR 2.2–3.108]	-
	Spike recovery (%)	-	125.3 [IQR 74.1–142.6]	129.9 [IQR 99.1–137]	96.7 [IQR 78.3–147.1]	108.6 [IQR 85.9–121.7]	113.8 [IQR 85.6–133.5]
	*P* (for % spike recovery)		0.08	0.043^*^	0.043^*^	0.043^*^	<0.001^*^
Healthy (*n* = 5)	Chromogenic assay						
	Endotoxin content (EU/ml)	0	0 [IQR 0–0.03]	0.031[IQR 0–0.04]	0.258 [IQR 0.109–0.267]	0.944 [IQR 0.636–1.434]	-
	Spike recovery (%)	-	0 [IQR 0–60.8]	31.5 [IQR 0–40.0]	51.5 [IQR 21.8–53.4]	37.7 [IQR 25.4–57.4]	34.0 [IQR 4.7–53.9]
	Turbidimetric assay						
	Endotoxin content (EU/ml)	0 [IQR 0–0.013]	0.071 [IQR 0.061–0.12]	0.151 [IQR 0.141–0.174]	0.532 [IQR 0.503–0.616]	2.132 [IQR 2.108–2.46]	-
	Spike recovery (%)	-	142.4 [IQR 95.1–212.5]	146.4 [IQR 127–173.4]	102.2 [IQR 99.9–123]	84.2 [IQR 82.6–101.3]	114.0 [IQR 98.3–145.4]
	*P* (for % spike recovery)		0.043^*^	0.043^*^	0.043^*^	0.043^*^	<0.001^*^

*HD* haemodialysis, *EU/mL* endotoxin units per millilitre

* denotes statistical significance *P* <0.05

### Comparison of assay error between turbidimetric and chromogenic LAL assay

**Fig. 1:**
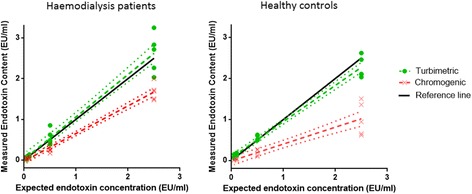
Comparison plots showing linear regression lines (*hashed lines*) for measured versus expected endotoxin content in spiked samples for HD patients and healthy controls with the chromogenic and turbidimetric assay. *Dotted lines* represent 95 % confidence intervals for linear regression lines. Each data point represents a mean of 4–6 measurements for one subject). *Black reference line* indicates perfect agreement between measured and expected endotoxin content

Comparison plots of measured versus expected endotoxin content in spiked samples were constructed (Fig. 1). Correlation between measured and expected endotoxin was higher for the turbidimetric assay compared to the chromogenic assay for both HD patients (*r* = 0.962 vs. 0.939) and healthy subjects *(r* = 0.954 vs. 0.922). For HD patients, estimates of proportional error and constant error were lower for the turbidimetric assay (4.2 % and 0.016 EU/ml [−0.13 to 0.162]) compared to the chromogenic assay (32.2 % and −0.027 EU/ml [−0.094 to − 0.041]) (Table 2 and Fig. 1). In healthy subjects, overall assay error was similarly lower in the turbidimetric assay compared to the chromogenic assay (proportional error, 11.8 % vs. 59.0 %; constant error, 0.067 EU/ml [−0.011 to 0.145] vs. −0.011 EU/ml [−0.128 to 0.102]) (Table 2 and Fig. 1).

**Table 2:** Comparison of assay error of turbidimetric and chromogenic LAL assay using linear regression analyses

Population	Assay	Slope	Proportional error (%)	Y-intercept (constant error) [EU/mL]	Correlation coefficient
HD (*n* = 5)	Turbidimetric	1.042 [0.927–1.157]	4.2	0.016 [−0.130–0.162]	0.962
	Chromogenic	0.678 [0.625–0.731]	32.2	−0.027 [−0.094–0.041]	0.939
Healthy (*n* = 5)	Turbidimetric	0.882 [0.821–0.943]	11.8	0.067 [−0.011–0.145]	0.954
	Chromogenic	0.41 [0.320–0.500]	59	−0.011 [−0.128–0.102]	0.922

*HD* haemodialysis, *EU/mL* endotoxin units per millilitre

**Fig. 2:**
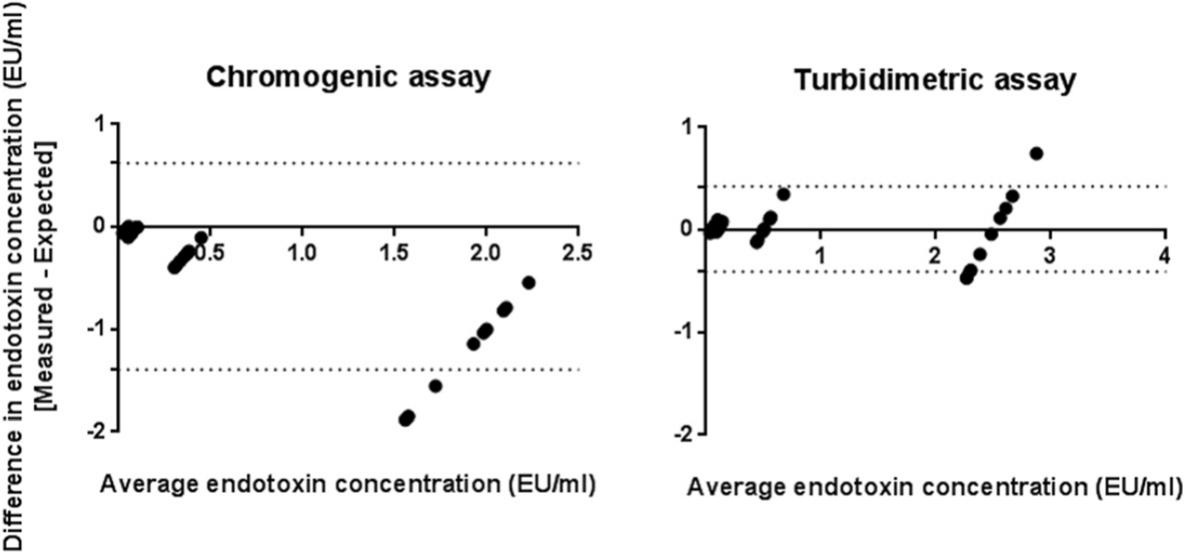
Bland-Altman analysis of measured versus expected spike recovery for the turbidimetric and chromogenic LAL assay in HD patients and healthy subjects (*dotted lines* represents 95 % limits of agreement)

Bland-Altman analysis was used to assess assay bias [14]. Bland-Altman plots for measured and expected endotoxin spike recovery the turbidimetric and chromogenic assay are shown in Fig. 2. Overall mean bias for the chromogenic assay was −0.384 EU/ml (95 % CI −0.219 to −0.549) and for the turbidimetric assay mean bias was 0.011 EU/ml (95 % CI 0.079 to −0.057). Sub-analysis of the HD cohort revealed mean bias for the chromogenic assay was −0.291 EU/ml (95 % CI −0.126 to −0.455) and significantly greater than the turbidimetric assay (0.049 EU/ml [95 % CI 0.162 to −0.064]; *p* < 0.001). Similarly, in healthy subjects there was greater bias with the chromogenic assay compared with the turbidimetric assay (mean bias −0.478 [95 % CI −0.776 to −0.180] vs. −0.021 EU/ml [95 % CI −0.102 to 0.061]; *p* < 0.001).

### Comparison of assay precision between turbidimetric and chromogenic LAL assay

The CV calculated using the onset reaction time for both cohorts was <10 % for both chromogenic and turbidimetric assays. CV calculated using the actual endotoxin content of the sample was <10 % for the turbidimetric LAL assay in pooled HD plasma samples spiked with high concentration of endotoxin (0.5 EU/ml), however in samples spiked with low concentration of endotoxin (0.05 EU/ml), CV was higher at 24.1 % suggesting imprecision of the turbidimetric assay at this low endotoxin concentration in HD patients. With the chromogenic assay, for samples spiked with high concentration of endotoxin (0.5 EU/ml), CV for HD patients and healthy subjects was ranged from 25.8 to 26.5 % suggesting high imprecision, CV for samples spiked with low amounts of endotoxin endotoxin (0.05 EU/ml) could not be defined because spike recovery was 0 % (Table 3).

**Table 3:** Coefficient of variation of turbidimetric and chromogenic assay in pooled uremic and non-uremic plasma

Concentration of endotoxin spike (EU/mL)		CV - onset reaction time (%)		CV - EU/ml (%)	
		0.05	0.5	0.05	0.5
Population	Assay				
HD (*n* = 2)	Turbidimetric	4.6	2.5	24.1	8.9
	Chromogenic	2.4	3.8	Undefined	25.8
Healthy (*n* = 2)	Turbidimetric	2.8	1.3	9.3	4.5
	Chromogenic	5.4	4.6	Undefined	26.5

In samples where spike recovery was 0 %, CV was undefined. *CV* coefficient of variation, *EU/mL* endotoxin units per millilitre

## Discussion

This is the first comparative study of two commonly used LAL detection techniques in haemodialysis patients. Two kinetic LAL assays utilising chromogenic and turbidimetric detection were compared directly in this study. Kinetic assays have significant advantages over older LAL techniques including the gel-clot and end-point method. Kinetic assays are more sensitive, able to quantify results over a wider range of endotoxin concentrations and benefits from having an automated process of analysis reducing the variation due to user technique [5]. In a recent review of endotoxin studies performed in dialysis patients [2], only one study utilised a kinetic assay [17], the remaining studies employed either the end-point technique [18, 19], the gel-point method [20, 21] or were not specified [22–26]. Despite their widespread availability and use, it is not known whether the turbidimetric or the chromogenic assay has superior performance in patients with end-stage kidney disease.

This study, using LAL reagents from the same manufacturer, has shown that the kinetic turbidimetric LAL assay is a more accurate and precise endotoxin detection tool compared to the chromogenic assay in HD patients and is able to detect endotoxin over a wide range of different endotoxin concentrations (0.05 –2.5 EU/ml). Additionally, this investigation showed that uraemic solutes that accumulate in patients with kidney failure do not appear to have any greater significant interference on the LAL assay than non-uraemic plasma.

Endotoxin spike recovery with the chromogenic assay was significantly inhibited by plasma from HD patients and healthy controls despite attempts to remove interfering factors from samples with dilution and heat treatment. Spike recoveries with the chromogenic were consistently below the minimum requirement of 50 % for healthy controls and overall median spike recovery was borderline acceptable at 53.8 % for the HD cohort. In comparison, median spike recovery for the turbidimetric assay was 113.8 %, well within the industry specified 50–200 % requirement [13]. The magnitude of assay error and bias was also greater for the chromogenic assay for both HD and healthy control subjects. Assay precision in this study was assessed using the coefficient of variation calculated from both the onset reaction time and the endotoxin content of the sample. There is a lack of industry guidance on which variable should be used to calculate the CV, depending on the manufacturer either the onset reaction time or the endotoxin read-out measurement can be used [16]. It has been suggested that CV calculated using the endotoxin content of samples is a more rigorous measure of assay precision [16]. Using the onset reaction time, CV was <10 % for both the turbidimetric and chromogenic LAL assay. However, CV calculated using the endotoxin content of the sample showed that the turbidimetric assay was more precise than the chromogenic assay. But in low levels of endotoxemia (≤0.05 EU/ml), assay imprecision was higher (CV −9.3 to 24.1 %) and care should be taken at interpreting samples containing low concentration of endotoxins.

Turbidimetric assays have the advantage of being more economical and a historical comparative study in non-uraemic plasma favoured the turbidimetric over the chromogenic assay because of the interfering effect of chromogenic substrates on the kinetics of the LAL reaction [27]. For end-point LAL assays, the chromogenic assay may suffer from interference as plasma and serum samples can absorb light at the measured 405 nm wavelength, potentially interfering with the results [28].

The LAL assay has been extensively used for the detection of endotoxin in pharmaceutical products, however its use in blood has been a matter of intense debate [29, 30]. Currently the LAL assay is not licensed by the Food and Drug Administration for ‘detecting endotoxemia in man’. This decision was heavily influenced by studies conducted by Stumacher [31] in 1973 and Elin in 1975 [32] demonstrating lack of concordance between endotoxemia and bacteraemia [29]. However, others have debated the validity of trials which measured the correlation of the LAL assay with positive culture bacteraemia as a ‘gold-standard’ [29], which is well known to have a low sensitivity for identifying patients with sepsis [33].

Due to current limitations with the LAL assay, a number of different novel detection assays have evolved over the last few decades including the recombinant factor C assay (rFC), the Endotoxin Scattering Photometry (ESP) assay and the Endotoxin Activity Assay (EAA) – a bioassay based on neutrophil activation by complement opsonised immune complexes of LPS [34]. No studies on the use of the rFC assay in human blood have been published to date. The ESP assay is a relatively novel detection system with its efficacy examined in a small number of trials [35–37]. The EAA is an FDA-approved blood endotoxin detection assay, however the EAA provides a read-out of a patient’s neutrophil oxidative chemi-luminescent response to LPS-antibody complexes rather than the direct endotoxin content present in the sample [34]. A recent investigation found a poor dose–response between blood spiked with control standard endotoxin and EAA activity [7]. Comparative studies between the optimum LAL assay and these novel detection assays would be useful.

The strengths of our study includes our meticulous attention to pre-analytical factors such as careful selection of blood sampling accessories by testing apparatus for contamination and interfering factors. No blood samples were obtained from tunnelled dialysis catheters to reduce the risk of contamination by bacterial biofilms [38]. Blood sampling and processing was performed rapidly to reduce the risk of endotoxin inactivation in untreated plasma [11]. The United States Pharmacopoeia states that in kinetic LAL testing, test samples need to be verified to be free of assay interfering factors by obtaining a spike recovery between 50 and 200 % from a positive product control (PPC) for an endotoxin measurement from a sample to be considered valid [13]. The PPC is a duplicate of the sample spiked with LPS at a concentration that lies near the mid-point of the standard curve *after* dilution and heat treatment. In this study, plasma samples were spiked with several different quantities of LPS *prior* to dilution and heat treatment which enables us to examine the ability of the assays to retrieve LPS from plasma containing wide range of different concentrations of endotoxin. Spiking samples before dilution and heat treatment to measure spike recovery also more closely resemble the process experienced by naturally occurring endotoxins present in the sample.

The limitations of this study include the relatively small number of participants and the use of control standard endotoxin derived to assess spike recovery. Standard endotoxins are derived from E.coli and are usually stabilised in preservatives such as lactulose and polyethylene glycol which may behave differently in vivo compared to naturally occurring endotoxin. However, natural endotoxins are difficult to standardise and many other published studies also use control standard endotoxin to assess recovery or immune response [10, 11, 27, 39–41]. The incubation times for samples spiked with endotoxin were not controlled due to the large number of samples that were processed, although laboratory analyses of spiked samples was carried out as soon as possible. It is unclear whether different incubation times can affect spike recovery. However since the same spiking procedure was used throughout the whole study by the same operator, incubation times would have been broadly similar between samples. We did not investigate whether the endotoxin measured in some of the baseline samples may have been due to (1 → 3) β-D glucan interference, which is well known to have the ability to activate the LAL assay via the factor G pathway [42], since the LAL assay used in this study was not modified to be insensitive to factor G activation. However, LAL is nearly 1000-times more sensitive to endotoxins than (1 → 3) β-D glucan [43] and contamination with (1 → 3) β-D glucan can usually be recognised by significant enhancement of spike recoveries [42], which was not seen in this study. Only one form of blood pre-treatment method (1:10 dilution and heat treatment at 70 °C for 15 min) was used in this study to remove interfering factors therefore the findings of this study may not be applicable if other blood pre-treatment methods were used. We did not explore different dilution or heating conditions, however the plasma pre-treatment conditions selected for this study were based on an extensive review by Hurley [11] and it is likely that the pre-treatment process for removal of interfering factors was sufficient since the overall spike recovery for the turbidimetric assay was >100 %. Our findings could also be criticised in that, since we compared chromogenic and turbidimetric assays using LAL reagent from one manufacturer only, our conclusions may not apply with use of reagents from other manufacturers. However the materials and methods described by the manufacturer are FDA approved for the testing of endotoxin prior to medical product release. As such, each batch is assessed and certified by the company and should be directly comparable with similar products from other manufacturers. Nevertheless, we suggest that assessment with reagents from alternative manufacturers is required before more general conclusions can be drawn.

## Conclusions

In summary, the association of endotoxemia with inflammation in dialysis patients needs to be confirmed with an accurate endotoxin detection assay to increase understanding of the potential harmful effects of chronic endotoxemia in this population and to develop endotoxin-lowering interventions. The study findings suggest that the kinetic turbidimetric LAL assay is a more accurate and precise assay compared to the chromogenic LAL assay, though these findings should be verified using LAL reagents from other sources.

## Abbreviations

CRP, C-reactive protein; CV, coefficient of variation; EAA, endotoxin activity assay; ESKD, end-stage kidney disease; esp, endotoxin scattering photometry; EU, endotoxin units; FDA, food and drug administration; HD, haemodialysis; LAL, limulus amebocyte lysate assay; LPS, lipopolysaccharide; PPC, positive product control; rFC, recombinant factor C assay
